# Supplementary figures and images for: Biallelic Loss of 7q34 (TRB) and 9p21.3 (CDKN2A/2B) in Adult Ph-Negative Acute T-Lymphoblastic Leukemia
Source: Int J Mol Sci. 2024 Sep 29;25(19):10482. doi: 10.3390/ijms251910482 (PMC11477120; doi:10.3390/ijms251910482)

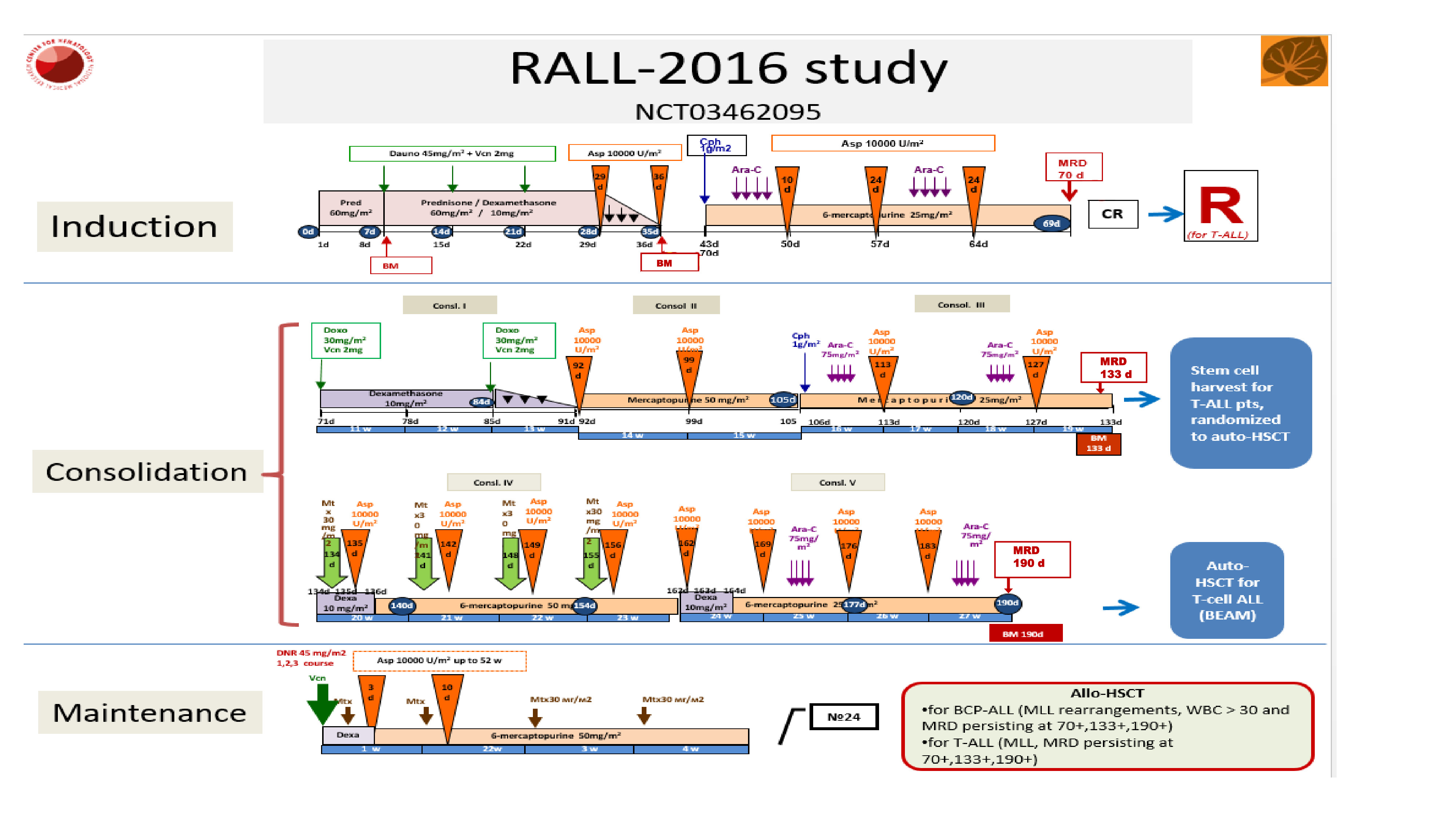

Supplement: Supplementary file 1 [file ijms-25-10482-s001.zip › Figure S1 R-ALL-2016 scheme.jpg]
